# Advances in optical imaging for pharmacological studies

**DOI:** 10.3389/fphar.2015.00189

**Published:** 2015-09-11

**Authors:** Alicia Arranz, Jorge Ripoll

**Affiliations:** ^1^Department of Cell Biology and Immunology, Center for Molecular Biology “Severo Ochoa”, Spanish National Research Council, Madrid, Spain; ^2^Department of Bioengineering and Aerospace Engineering, Universidad Carlos III of Madrid, Madrid, Spain; ^3^Experimental Medicine and Surgery Unit, Instituto de Investigación Sanitaria del Hospital Gregorio Marañón, Madrid, Spain

**Keywords:** bioluminescence, planar fluorescence imaging, fluorescence molecular tomography, optoacoustics, multispectral optoacoustic tomography, multispectral imaging, hybrid systems, data processing

## Abstract

Imaging approaches are an essential tool for following up over time representative parameters of *in vivo* models, providing useful information in pharmacological studies. Main advantages of optical imaging approaches compared to other imaging methods are their safety, straight-forward use and cost-effectiveness. A main drawback, however, is having to deal with the presence of high scattering and high absorption in living tissues. Depending on how these issues are addressed, three different modalities can be differentiated: planar imaging (including fluorescence and bioluminescence *in vivo* imaging), optical tomography, and optoacoustic approaches. In this review we describe the latest advances in optical *in vivo* imaging with pharmacological applications, with special focus on the development of new optical imaging probes in order to overcome the strong absorption introduced by different tissue components, especially hemoglobin, and the development of multimodal imaging systems in order to overcome the resolution limitations imposed by scattering.

## Introduction

Whole-body optical *in vivo* imaging approaches are valuable tools that enable the study of animal models of human diseases, reducing the number of animals required for experimentation and providing essential information in pharmacological studies. Depending on the physical principle providing image contrast, we find techniques based on light generation, such as bioluminescence or fluorescence imaging, or based on light absorption, such as optoacoustics. All these methodologies enable *in vivo* imaging of molecular and cellular processes with high sensitivity and have gained great popularity over the past decade mainly because of their safe and straightforward use due to the employment of non-ionizing wavelengths, and their cost-effectiveness compared with other imaging technologies (such as positron emission tomography, PET, or magnetic resonance imaging, MRI; Ntziachristos et al., 2007; Stuker et al., 2011b).

On the other hand, one of the main problems of optical *in vivo* technologies is dealing with the scattering and absorption properties of tissue (Boas et al., 2011; Ripoll, 2012): scattering is responsible for the loss of light directionality (and therefore a loss in resolution by consequently blurring the image), while the presence of high absorbers (such as melanin and blood) results in a reduction of light intensity (decreasing the signal to noise ratio dramatically in the visible range; Ripoll, 2012).

The most effective way to overcome the loss of signal intensity due to absorption is to employ excitation and emission wavelengths in the near-infrared optical imaging window (between 700 and 900 nm, approximately), where the main tissue constituents (hemoglobin, melanin, water, and lipids) absorb the least (Ntziachristos et al., 2005; Jacques, 2013). On the other hand, if one wishes to account for the effects of high scattering in light propagation within tissues in order to obtain a 3D image or quantitative information (note that location, probe concentration, and probe size are strongly interdependent), one needs to introduce a physical model of light propagation within complex media such as a living organism. Once this model is in place, a numerical inversion of this model (what is termed, “solving the inverse problem”) is needed in order to obtain a 3D image providing the spatial distribution of probe concentration. Depending on the algorithm we use to reconstruct an image we will be able to recover probe size, position and concentration with varying accuracy. How this issue is addressed clearly distinguishes the different imaging approaches in optical *in vivo* imaging into the following three categories: (1) planar optical imaging, (2) optical tomography, and (3) optoacoustic tomography.

In this review we discuss the latest advances of optical *in vivo* imaging as a tool in pharmaceutical studies, addressing the different approaches that are being developed in order to overcome the strong absorption introduced by hemoglobin and the ill-posedness introduced by scattering, either through the use of multimodal imaging or photoacoustic tomography, or by developing new probes or proteins more adequate for *in vivo* imaging in deep tissues.

## Planar Optical Imaging

Planar optical imaging techniques are by far the most common, mainly due to their simplicity of use and low cost. Two planar imaging modalities are available, depending on the light source generation: Bioluminescence and Fluorescence. In both cases a high sensitivity camera (CCD mainly) coupled to a high numerical aperture camera objective takes a single long exposure image, in the case of fluorescence using appropriate band-pass filters. In what follows we detail recent advances and applications in both modalities.

### Bioluminescence *In Vivo* Imaging

Bioluminescence imaging is based on the oxidation of a substrate (luciferin) mediated by an enzyme (luciferase), being the most commonly used the luciferase originated from the North American firefly (*Photinus pyralis*). The firefly luciferase requires ATP and magnesium to catalyze the reaction that leads to the emission of light, which ranges from 530 to 640 nm, depending amongst other factors on the pH, polarity of the solvent, and the microenvironment of the enzyme (Li et al., 2013). Note how this emission falls within the portion of the visible spectrum where hemoglobin is strongly absorbing.

Since the firefly luciferase was cloned (de Wet et al., 1985), the *luc* gene has extensively been used in gene regulation studies. Bioluminescent probes have also been engineered in order to detect specific enzymatic activities. These probes are designed in such a way that the luciferin is “caged” and this conjugate has to be cleaved by an enzymatic activity (i.e., proteases such as caspases). Once cleaved, the luciferin can be oxidized by the luciferase and the signal is released (Li et al., 2013).

Techniques based on bioluminescence detection have largely been used for molecular biology assays in laboratories worldwide. Accordingly, bioluminescence has also been a reference method for *in vivo* imaging. Its main advantage is the absence of background signal (the commonly used cell or animal models do not express luciferase and therefore there is no “auto-bioluminescence”), which leads to a high specificity of the detected signals and an elevated signal-to-noise ratio. This has resulted in an impressive expansion of bioluminescence *in vivo* imaging applications for studies in cancer biology, inflammation, and infection, amongst others (Edinger et al., 2002; Andreu et al., 2010; Luker and Luker, 2011; Luwor et al., 2015). However, researchers using bioluminescence *in vivo* imaging have to deal with problems derived from the complexity of the luciferase-luciferin reaction and the effects of light propagation in living tissues. Regarding the luciferase-luciferin reaction, both substrate and co-factors (ATP, oxygen and magnesium) are required for the reaction to take place and therefore the limitation of any of them may result in altered readouts that are not a real representation of luciferase activity (Sadikot and Blackwell, 2005). There have also been significant efforts toward the development of bioluminescence tomography (BLT) approaches, requiring the prior knowledge of one of the parameters or the number of sources in order to produce a 3D image (Liu et al., 2010).

### Fluorescence *In Vivo* Imaging

After a fluorescent agent is excited with a light source, fluorescence is emitted isotropically as a consequence of a radiative transition from an excited singlet state to a singlet state of lower energy (typically the ground state) following Stoke’s Law (Sauer et al., 2011). Even though fluorescence has been extensively used in microscopy for over a century to study molecular and cellular processes (Masters, 2009), it has not been until this past decade that its use for *in vivo* small animal imaging became significant (Mahmood et al., 1999; Weissleder et al., 1999; Ntziachristos et al., 2005). The high sensitivity offered by this technique and the latest advances in fluorescence labeling have also promoted its relatively recent incursion in non-invasive *in vivo* imaging. Both planar and three-dimensional fluorescence imaging methods *in vivo* are now commonly used in pre-clinical research.

In order to acquire a fluorescence image, either as part of a tomographic data set or a single planar image, one requires an excitation source as close as possible to the excitation maximum of the fluorophore being used, if possible within the near infrared optical imaging window. The use of this excitation wavelength, however, will not only excite specifically the fluorophore but will generate non-specific fluorescence from several components present in tissue, generating what is termed “auto-fluorescence,” reducing the signal to background ratio (i.e., the contrast in the image). One way to reduce this problem is performing several spectral measurements with different excitation/emission pairs and unmixing the specific signal of the fluorophore from the un-specific signal of the surrounding tissue (Xu and Rice, 2009).

**FIGURE 1:**
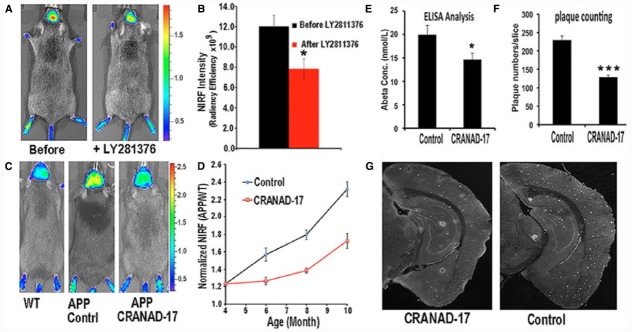
*****In vivo*** monitoring of therapeutic effect of drug treatment in Alzheimer’s disease.** Application of CRANAD-3 for monitoring therapeutic effects of drug treatments. **(A)** *In vivo* imaging of APP/PS1 mice with CRANAD-3 before and after treatment with the BACE-1 inhibitor LY2811376. **(B)** Quantitative analysis of the imaging in A (*n* = 4). **(C)** Representative images of 4-month-old APP/PS1 mice after 6 months of treatment with CRANAD-17. (Left) Age-matched WT mouse. (Center) Control APP/PS1 mouse. (Right) CRANAD-17—treated APP/PS1 mouse. Note that the NIRF signal from the CRANAD-17—treated APP/PS1 mouse (Right) is lower than the signal from the non-treated control APP/PS1 mouse (Center). **(D)** Quantitative analysis of the imaging in C (*n* = 5). (E) ELISA analysis of total Aβ40 from brain extracts. **(F)** Analysis of plaque counting. **(G)** Representative histological staining with thioflavin S. (Left) CRANAD-17—treated mouse. (Right) Control. *P < 0.05, **P < 0.01, ***P < 0.005. From Zhang et al. (2015b).

With respect to recent pharmacological studies, Zhang et al. (2015b) make use of planar fluorescence molecular imaging to monitor therapy in murine models of Alzheimer’s disease. In particular, the authors verify the feasibility of using CRANAD-3 for monitoring therapy, and use it to monitor the therapeutic effect of CRANAD-17, a curcumin analog for inhibition of Aβ cross-linking (see Figure 1).

## Diffuse Optical Tomography and Fluorescence Molecular Tomography

In order to account for the effect of scattering when imaging tissues with light, diffuse optical tomography (DOT) was developed, based on scanning a point source over the sample and measuring the intensity of the diffuse light either by fibers or with a camera focused onto the surface (see Arridge, 1999, for a review on this subject). With its first applications being targeted toward breast cancer (see, for example Ntziachristos et al., 2000) its use in small animal imaging came with the development of fluorescence molecular tomography, first published in 2002 (Ntziachristos et al., 2002), in the context of molecular imaging by employing an activatable probe to image protease activity in an *in vivo* mouse model of glioblastoma. Since this first publication in 2002 there have been several developments and applications, mainly in tumor biology (Ntziachristos et al., 2004; Deliolanis et al., 2006; Montet et al., 2007; Kossodo et al., 2010; Hensley et al., 2012) and inflammation studies (Martin et al., 2008; Kang et al., 2014; Thomas et al., 2015), amongst others.

Apart from suffering from auto-fluorescence in a manner similar to planar fluorescence imaging, DOT and FMT provide no anatomical information and therefore benefit from its combination with measurements provided by other imaging systems such as X-ray computed tomography (CT) or MRI, issue which we will discuss at the end of this review. Additionally, the prior knowledge of anatomical features and optical properties significantly improves image quality and quantitation, as will be discussed later.

## Optoacoustic *In Vivo* Imaging

Being based on the emission of sound after a transient increase in volume due to light absorption, the photoacoustic effect may be used to image in 3D the location and relative concentration of fluorescence probes using advanced acoustic transducers and light sources. Termed Optoacoustic or Photoacoustic imaging, it circumvents the “blurring” caused by scattering on the visible wavelengths by measuring the acoustic wave generated, which suffers several orders of magnitude less scattering, resulting in an increased penetration depth with no significant loss of signal to noise. In order for this approach to be implemented and transient volume changes generated, we need to use pulsed lasers and then record the ultrasound wave generated by the localized absorption of this pulse of light by the tissue. Recording this ultrasound wave at several locations simultaneously, we may make use of tomographic methods to recover a 3D image (Wang et al., 2003). When multispectral methods are used, such as in multispectral optoacoustic tomography (MSOT), different fluorophores may be separated and their relative concentration quantified (Laufer et al., 2007; Ma et al., 2009; Tzoumas et al., 2014), underlying the use of MSOT for quantitative and highly specific *in vivo* imaging. Additionally, since hemoglobin is a strong absorber, optoacoustic tomography may also be used for resolving vascular structures and quantifying oxygen saturation and blood volume (Lao et al., 2008; Hu and Wang, 2010). The high resolution of MSOT—approximately ∼100 μm and in some cases even better [∼40 μm resolution was shown in Ma et al. (2009)], good anatomical information, and quantitative 3D images are the reason why this approach is becoming widespread in pharmacological studies.

**FIGURE 2:**
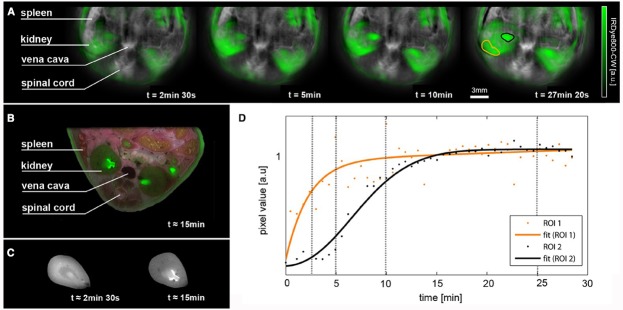
**Pharmacokinetic ***in vivo*** imaging using MSOT. (A)** Time series of images visualizing the biodistribution of IRdye800 in green on logarithmic scale overlaid on the vasculature. Both channels are the result of spectral unmixing. **(B)** Cryoslice image after approximately 15 min with overlaid fluorescence as a verification of the MSOT results. **(C)** A comparison of fluorescence distribution in the kidneys of mice sacrificed after approximately 2 min 30 s after injection and 15 min after injection. Note the changes in distribution similar to the time series shown in **(A)**. **(D)** Temporal evolution of signal (each normalized to their smoothed maxima) in the regions of interest highlighted in the rightmost image, orange showing a region in the renal cortex that displays early and steep signal pickup and black indicating a region in the renal pelvis where probe accumulation is delayed and has a smoother profile. Time points of the images in **(A)** are marked using vertical lines. From Taruttis et al. (2012).

One application of MSOT to pharmacological studies which show extremely high impact is the use of MSOT to follow pharmacokinetics *in vivo* (Kossodo et al., 2010; Razansky et al., 2011, 2012; Bednar and Ntziachristos, 2012; Taruttis et al., 2012). Figure 2 shows an example of the potential of MSOT, where a time series of images visualizing *in vivo* the biodistribution of IRdye800 and vasculature are shown. This study shows how the spatially localized temporal evolution of drug delivery may be imaged in real time.

One of the drawbacks of optoacoustic tomography is its lower sensitivity when compared to pure fluorescence measurements and the difficulty of imaging in organs that present high acoustic contrast or high impedance mismatch, such as the lungs. Another drawback is that the signal generated is proportional to the light intensity that has been absorbed locally and thus decreases for deeper tissues. Even though the lack of knowledge on the precise light distribution within the subject precludes this technique from being fully quantitative, the development of advanced inverse methods and imaging approaches are constantly improving the quantitative nature of MSOT (Razansky et al., 2011).

## Latest Advances to Improve Quantification and Resolution

Once we have covered the main optical imaging approaches, we now will present the most recent advances to improve these imaging techniques either by changing the emission spectra of the probes or by including anatomical information and thus reducing the ill-posed nature of the inverse problem.

### Avoiding Absorption in Living Tissues: Moving Toward the Near Infra-Red

As mentioned previously, working with wavelengths in the near infra-red (NIR), in particular in the 700–900 nm window, reduces the amount of light absorbed in tissues by ∼3 orders of magnitude when compared to the visible spectrum. Due mainly to hemoglobin absorption and considering that the emission peak of the native firefly luciferase is in the range of ∼562 nm, its detection is mainly limited to the surface. Great efforts have been focused on obtaining mutated versions of luciferase enzymes leading to red-shifted emission wavelengths, with emission peaks above 600 nm (Branchini et al., 2010a; Stepanyuk et al., 2010; Mezzanotte et al., 2011; Wang et al., 2013). In order to obtain emitted light with longer-wavelengths, considerable effort has also been devoted to the development of analogs of the substrate (luciferin), such as aminoluciferins (Mofford et al., 2014) or selenium analogs (Conley et al., 2012). Other developments have been bioluminescence resonance energy transfer (BRET) conjugates, consisting on using the emitted bioluminescence light as excitation for fluorescent molecules. The use of these conjugates results in a final emitted light above 700 nm (Branchini et al., 2010b), although it has been discussed that they may alter the cellular uptake properties of the substrate (Conley et al., 2012).

In the case of fluorescence, an impressive development of new NIR fluorescent agents has taken place in recent years with excitation maxima above 650 nm, allowing the use of excitation sources and emission spectra within the optical window of the spectrum, where blood absorption is reduced to a minimum (Ntziachristos et al., 2005; Jacques, 2013). Researchers can now benefit from a wide portfolio of near infra-red fluorescent (NIRF) probes designed to be non-targeted (non-specific used for imaging of perfusion or vascular leakage), targeted (such as fluorescent-conjugated antibodies, which recognize and bind specific ligands), or activatable (the fluorescent signal is quenched unless a specific enzymatic activity cleaves the probe). Moreover, different approaches have been followed to obtain NIRF proteins, reaching excitation maxima above 670 nm (Shcherbo et al., 2007; Shu et al., 2009; Filonov et al., 2012). Constructs for the expression of these proteins and recently developed transgenic mice (Diéguez-Hurtado et al., 2011; Tran et al., 2014) provide an excellent tool for *in vivo* imaging applied to biomedical and pharmaceutical studies.

With respect to optoacoustics, all advances in fluorescent probes are directly compatible with this methodology, since probes with high quantum yield by definition present high absorption properties. Additionally, optoacoustic imaging methods are also benefiting from new engineered acoustic probes based on metallic nanoparticles (mainly gold) which exhibit high absorption profiles (Bao et al., 2013; Vonnemann et al., 2014).

Finally, a very interesting and promising new development is the use of Cherenkov excited luminescence imaging (CELSI) to improve resolution and partially avoid the effect of absorption while exciting the fluorophores (Zhang et al., 2012, 2013b, 2015a). This approach makes use of Cherenkov emitted NIR light from collimated ionizing radiation generated in a linear accelerator (LINAC), a technique which could potentially be applied for imaging fluorescent markers deep in tissue with high resolution.

### Hybrid Systems

The combination of optical imaging modalities with structural imaging methods such as X-ray CT or MRI allows obtaining anatomical information that can be used as prior data on the reconstruction algorithm to improve both resolution and sensitivity (Ale et al., 2012).

For example, FMT-MRI hybrid systems have been developed and used to analyze protease activity and tumor morphology in mouse tumor models (Davis et al., 2010; Stuker et al., 2011a) or metalloproteinase activity in mouse models of atherosclerosis (Li et al., 2014). FMT-XCT hybrid systems are also examples where we can make use of anatomical priors obtained from the geometric information provided by the XCT measurements in order to improve the 3D reconstruction of the fluorescence signal (Ale et al., 2012; Zhang et al., 2013a, 2014).

The technical complexity of these hybrid systems (for example, due to crosstalk between optical and MRI imaging) has led to the use of adapted animal holders which are compatible with different modality systems enabling sequential imaging (McCann et al., 2009).

## Conclusions and Future Outlook

A wide range of optical imaging modalities are available for *in vivo* imaging in small animals, representing an essential tool in pharmacological studies. Each modality, however, presents its own drawbacks, mainly due to the effects of absorption and scattering of light propagation in living tissues. As shown in Table 1, the selection of a technique will depend on the model used and the information that we want to obtain. For example, if high-throughput imaging is required, planar imaging approaches will be useful, with the consequence that no quantitative information or depth location may be inferred (see Table 1). If quantitative imaging and probe location is important, tomography is needed and FMT and similar approaches are a good option, reaching their full potential when combined with an anatomical imaging modality such as MRI or X-ray CT. As a quickly growing modality, optoacoustic tomography and in particular MSOT shows great potential, so far offering the best imaging resolution, but with the problems associated with ultrasound imaging such as high impedance mismatch in some organs such as the lungs and the need for a matching gels.

**TABLE 1:** **Comparison of different imaging modalities**.

**Technique**	**Resolution**	**Throughput**	**Pharmacokinetics**	**3D Info**
Bioluminescence	>5 mm*	High	No	No
Planar fluorescence	>5 mm*	High	No	No
FMT	1–2 mm	Medium	No	Yes
FMT/XCT	1 mm	Low	No	Yes
MSOT	0.1 mm	Low	Yes	Yes

*Resolution depends on depth location.

We believe that as more specific near infrared fluorescent probes and proteins with distinct spectral features, and specific nanoparticles for high and specific optoacoustic signal generation are generated there will be further improvement of the performance of the technologies covered in this review, opening opportunities for new applications. The combination of several imaging modalities, specifically if they include optical imaging approaches, will ensure the sensitivity and specificity that optical probes uniquely offer may reach their full potential as imaging agents for 3D quantitative imaging *in vivo*.

### Conflict of Interest Statement

The authors declare that the research was conducted in the absence of any commercial or financial relationships that could be construed as a potential conflict of interest.
